# Quantification of Lipoteichoic Acid in Hemodialysis Patients With Central Venous Catheters

**DOI:** 10.3389/fmed.2018.00308

**Published:** 2018-11-05

**Authors:** Amy Barton Pai, Adinoyi Garba, Paul Neumann, Alexander J. Prokopienko, Gabrielle Costello, Michael C. Dean, Sriram Narsipur

**Affiliations:** ^1^College of Pharmacy, University of Michigan, Ann Arbor, MI, United States; ^2^D'Youville College School of Pharmacy, Buffalo, NY, United States; ^3^Albany College of Pharmacy and Health Sciences, Albany, NY, United States; ^4^Center for Clinical Pharmaceutical Sciences, School of Pharmacy, University of Pittsburgh, Pittsburgh, PA, United States; ^5^SUNY Upstate Medical University, Syracuse, NY, United States

**Keywords:** lipoteichoic acid, catheter, dialysis, inflammation, biomarker

## Abstract

Hemodialysis patients with central venous catheters (CVCs) have chronic systemic inflammation, the source of which may be related to intraluminal bacterial biofilm. There is currently no non-invasive method to adequately evaluate intraluminal biofilm. Lipoteichoic acid (LTA) is a Gram-positive bacterial cell wall component that is spontaneously shed. The purpose of this study was to determine whether LTA could be quantified in biological samples and to evaluate potential relationships to markers of inflammation. Heparin-locked catheter aspirate was drawn from both the arterial and venous ports of each CVC prior to dialysis initiation. Venous blood from the dialysis circuit was collected 30 min after dialysis initiation. LTA was quantified in aspirate and plasma. Key markers of inflammation (interleukin-6, and hepcidin) and endothelial dysfunction (soluble vascular endothelial cadherin) were also determined in plasma samples. Catheter aspirate and systemic blood samples were obtained from 40 hemodialysis patients. The median (range) duration of catheter use was 130 (20–1635) days. Unexpectedly, median (range) plasma LTA concentrations (ng/mL) were significantly higher than catheter aspirate LTA concentrations [3.93 (0.25–15) vs. 2.38 (0.1–8.1), respectively, *p* = 0.01] in the majority (70%) of patients. Area under the receiver operator characteristic (ROC) curve showed good potential prognostic value of catheter aspirate LTA predicting systemic LTA concentrations with an area under the curve of 0.815 (95% CI, 0.68–0.95). A significant correlation was found between LTA and serum ferritin (*r* = 0.32, *p* = 0.04), however, there were no significant correlations between LTA and the other inflammation biomarkers assessed. LTA is quantifiable in aspirate and plasma of hemodialysis patients with CVCs and warrants further investigation to determine potential clinical application to intraluminal biofilm evaluation.

## Introduction

Despite active initiatives to start hemodialysis with permanent vascular access, tunneled central venous catheter (CVC) use remains high at dialysis initiation and infection continues to be the second leading cause of death in end-stage renal disease (ESRD) patients (1, 2). Hemodialysis patients with CVCs have significantly higher C-reactive protein and markedly lower serum albumin concentrations, both of which are biomarkers of inflammation with strong associations with mortality in the hemodialysis population (3, 4). Several studies have shown that CVCs are rapidly colonized after insertion with Gram-positive organisms that can form intraluminal biofilm which can be a source of inflammation if planktonic bacteria or biofilm fragments enter the systemic circulation (5). Bosma et al. evaluated 32 newly placed CVCs and showed that 30 days after catheter insertion, 100% had biofilm formation when examined by scanning electron microscopy. However, only 11 (34%) had positive cultures. These data show that culture screening methods are not reliable in detecting intraluminal biofilm and do not provide information about biofilm surface area coverage (6). A principal component of the Gram-positive bacteria cell wall is lipoteichoic acid (LTA) (7) which is a key mediator of inflammation in Gram-positive infections, activating NFκB via the toll-like 2 (TLR2) receptor resulting in production of pro-inflammatory cytokines. (8, 9) LTA is a transmembrane glycoprotein that is released from the bacteria cell wall and thus could be a potential biomarker to non-invasively assess catheter biofilm burden. Recently, semiquantitative measurement of LTA has been investigated as a bioassay to detect early Gram-positive blood stream infections (10). However, in hemodialysis patients and other populations requiring longer-term CVC use, inflammation induced by dissociated intraluminal biofilm fragments likely precedes infection (5). There are currently no published studies that have quantified LTA in the catheter aspirate and systemic circulation of patients with CVCs. A non-invasive marker of biofilm burden could be exploited as a surrogate endpoint for dialysis catheter studies (11). A validated biomarker of intraluminal biofilm could also be utilized to inform clinical decisions such as accelerating permanent vascular access placement, catheter removal/replacement or installation of anti-microbial lock solutions (11–13). This pilot, proof of concept study sought to determine whether concentrations of LTA in catheter aspirate and the systemic circulation of hemodialysis patients with CVCs could be quantified. Clinically relevant biomarkers of inflammation and endothelial permeability were also evaluated to explore potential associations with LTA concentrations.

## Materials and methods

The study was carried out in accordance with the recommendations of the Guidelines for Preparing Written Consent by the Institutional Review Board at Albany College of Pharmacy and Health Sciences. All subjects gave written informed consent in accordance with the Declaration of Helsinki. The protocol was approved by the Institutional Review Board at Albany College of Pharmacy and Health Sciences (protocol number 10-014). Eligible patients were adults > 18 years on hemodialysis for >3 months with tunneled CVCs.

### Sample collection

Heparin-locked catheter aspirate equal to the volume of the luminal dead space specified on each catheter port (between 1.2 and 2 mL) was drawn from both arterial and venous ports prior to dialysis initiation. Venous blood from the dialysis circuit was collected 30 min after dialysis initiation to evaluate the potential effect of dialysis shear stress on biofilm stability. Samples were aliquoted and stored at −80°C for later analysis.

### Biomarker assays

LTA concentrations in aspirate and plasma were measured according to manufacturer's instructions with a commercially available ELISA kit (MyBioSource, San Diego, CA, USA). To evaluate potential relationships between LTA and induction of inflammation, plasma levels of hepcidin, interleukin 6 (IL-6), and soluble vascular endothelium cadherin (sVE cadherin) were measured by commercially available ELISA kits. The hepcidin ELISA kit (DRG Instruments, GmbH, Marburg, Germany), IL-6 ELISA kit (Enzo Life Sciences, Farmingdale, NY), and sVE cadherin ELISA kit (R&D Systems, Minneapolis, MN) were all used according to the manufacturer's protocols.

### Statistical analysis

Data are reported as median (range) unless otherwise noted. All data were tested for normality (Shapiro-Wilk's test). Correlation between continuous variables was examined by Spearman's rank correlation coefficient. Linear regression was performed to evaluate the relationship between catheter and systemic LTA with catheter LTA as the dependent variables. The potential diagnostic value of catheter aspirate LTA concentration to predict systemic LTA concentration was evaluated by area under the receiver operator characteristic (ROC) curve analysis. Analyses were performed using in R Statistical Software (Version 3.1.0) *P*-values < 0.05 were considered statistically significant. All probabilities were two-tailed.

## Results

**Figure 1:**
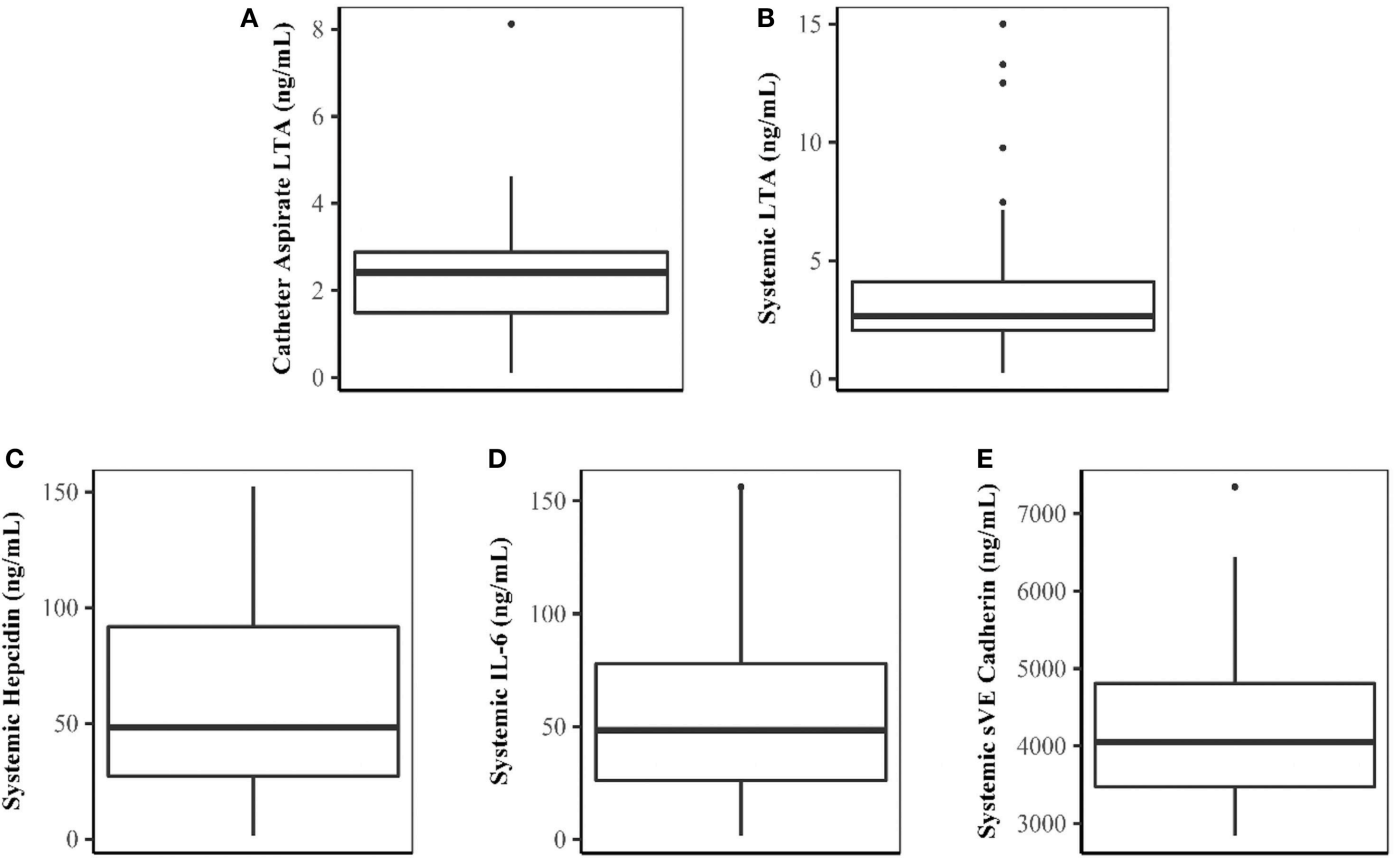
Concentrations of LTA and Biomarkers of Inflammation and Endothelial Dysfunction. **(A–E)** Represent catheter aspirate LTA, systemic LTA, hepcidin, IL-6, and sVE cadherin concentrations, respectively. Concentrations are expressed in ng/mL.

Forty patients completed the study. Demographic characteristics of the study population are shown in Table 1. LTA was quantifiable in both catheter aspirate and the systemic circulation (Figures 1A,B, respectively). Linear regression analysis demonstrated a relationship between catheter aspirate LTA concentrations and systemic LTA concentrations (*R*^2^ = 0.26, *p* = 0.0003). Concentrations of hepcidin, IL-6 and sVE cadherin were elevated (Figures 1C–E, respectively), however, no correlation was observed with catheter or systemic LTA concentrations. A significant positive correlation was observed with catheter aspirate LTA and serum ferritin, a known acute phase reactant (*r* = 0.32, *p* = 0.04).

**Table 1:** Study population demographic and baseline characteristics.

**Variable**	**Mean ±SD (range)**
Age (years)	54.85 ± 15.90 (23–86)
Catheter duration (days)	258 ± 345 (median: 130)
Ferritin (ng/mL)	758 ± 436 (median: 839)
Albumin (g/dL)	3.6 ± 0.5 (2.6–5.0)
LTA catheter aspirate (ng/mL)	2.38 ±1.47 (0.1–8.1)
LTA systemic (ng/mL)	3.93 ± 3.39 (0.25–15)
Hepcidin (ng/mL)	60.14 ± 42.26 (1.48–152.2)
	^*^^a^28.1 (20.5–66)^25^
IL-6 (ng/mL)	56.07 ± 40.43 (1.56–156.20)
	^*^0.88–2.2^26^
sVE cadherin (ng/mL)	4268.14 ± 1040.84 (2839–7346)
	^*^range: 1375-4294^27^

* Reported concentrations in healthy controls (14, 15).

a *geometric mean (5-95% range)*.

Given the observed positive correlation between aspirate catheter LTA concentrations and systemic LTA concentrations, a sub-analysis was performed to evaluate patients who had higher systemic LTA concentrations after initiating dialysis compared to measured aspirate catheter LTA concentrations (*n* = 27, 70%). A strong, positive correlation between catheter and systemic LTA concentrations was observed among these patients (*r* = 0.76, *p* < 0.0001). Linear regression analysis also demonstrated a strong relationship between catheter aspirate LTA concentrations and systemic LTA concentrations (*R*^2^ = 0.58, *p* = 0.0003). ROC analysis showed that the level of catheter aspirate LTA had a good potential prognostic value to predict systemic LTA concentrations with an area under the curve of 0.815 (95% Confidence Interval, 0.68–0.95).

## Discussion

This study is the first to show that LTA is detectable in aspirate from CVCs and in the systemic circulation after hemodialysis initiation. Previous studies have shown that only a small percentage of catheter tip or aspirate cultures are positive when biofilm presence is confirmed by scanning electron microscopy. (8) Thus, new methods need to be evaluated to non-invasively assess intraluminal biofilm. It was an unexpected finding that LTA concentrations were higher in the systemic circulation after 30 min of dialysis in most patients given the expected relatively high density of intraluminal biofilm in the catheter. Although *Staphylococcus* sp. biofilms have inherent viscoelasticity properties that allow them to resist detachment under conditions of shear stress, this has not been evaluated for high blood flow rates that simulate shear stress within dialysis catheters. (16). It is well documented that hemodialysis patients with CVCs have markedly higher inflammatory profiles compared to patients with grafts and fistulas (17). LTA binds to toll-like receptor TLR2 which promotes transcription of pro-inflammatory cytokines such as IL-6, which can stimulate hepcidin production, via intracellular signaling pathways that include reactive oxygen and nitrogen species (9, 18). We have previously shown that TLR2 activation by LTA from *Staphylococcus aureus* has deleterious effects on endothelial cell adhesion and increases permeability (19). Cadherin is critical in maintaining the integrity of intercellular junctions creating a restrictive endothelial barrier (20). Soluble vascular endothelial cadherin has been shown to be a prognostic marker in inflammatory disease states (20, 21). We found the mean of measured plasma markers of inflammation (IL-6, hepcidin) and endothelial barrier dysfunction (sVE cadherin) to be much higher than values reported for healthy subjects (14, 15, 22). However, there was significant interpatient variability was no correlation with LTA in catheter aspirate or plasma in was found in this small pilot study. However, a positive correlation was shown with catheter aspirate LTA and ferritin which is an acute phase reactant that is elevated in diseases with chronic inflammation including chronic kidney disease (23).

These data should be considered in the context of several limitations. This pilot study was a small, proof of concept study and these findings need to be corroborated in a larger study, compared to other permanent vascular access types and confirmed with objective biofilm assessment with scanning electron microscopy. Dialysis is known to induce inflammation which may have contributed to the large interpatient variability observed in the inflammatory biomarkers measured. It should be noted that measuring bacterial cell wall components by ELISA does not have specificity for particular bacterial species and would be limited in guiding empiric antimicrobial therapy section. However, if a biomarker obtained from catheter aspirate samples can indicate relative intraluminal biofilm burden and/or biofilm instability, this could provide clinicians with additional information to guide treatment decisions, including antimicrobial lock solutions and salvage antibiotic therapy versus catheter removal (11). A recent consensus statement a multidisciplinary panel comprised of representatives from the American Society of Nephrology and Food and Drug Administration cited the several areas where high-priority studies regarding dialysis catheter endpoints are urgently needed including; “Studies to assess whether CVC intraluminal colonization in asymptomatic patients on dialysis precedes most bloodstream infections and whether it may be a reliable surrogate outcome for future studies” (11). Thus, if validated, catheter aspirate LTA concentrations or other non-invasive biofilm biomarkers could potentially be exploited for both clinical and research use in the future. Follow up studies should also utilize microbiologic screening and metagenomic techniques to further elucidate the hemodialysis catheter microbiome communities (24).

## Conclusion

In summary, the Gram-positive cell wall component LTA is quantifiable in hemodialysis patients with CVCs and should be studied further to evaluate the potential clinical utility of LTA as a biomarker of intraluminal biofilm.

## Data availability

Raw data supporting the conclusions made in this manuscript are available upon request to qualified researchers.

## Author contributions

AB and AG: Study conception and design. AB, AP, AG, and PN: Acquisition of data. AB, AG, AP, SN, GC, and MD: Analysis and interpretation of data. AB, AP, GC, MD, and PN: Drafting of manuscript. AG and SN: Critical revision.

### Conflict of interest statement

The authors declare that the research was conducted in the absence of any commercial or financial relationships that could be construed as a potential conflict of interest. The reviewer JMJ and handling Editor declared their shared affiliation.
